# Synthesis of reusable cement materials through photochemical modification of marble powder for composite structures

**DOI:** 10.1016/j.heliyon.2024.e41570

**Published:** 2024-12-30

**Authors:** Ali Zia Noor, Muhammad Atif, Sadia Bibi, Muhammad Burhan Sharif, Amjad Ali

**Affiliations:** a Chemistry Department, University of Education Lahore (Vehari Campus), Punjab, Pakistan; b Department of Civil Engineering, University of Engineering and Technology, Lahore, Punjab, Pakistan; c Department of Physics, University of Okara, Okara, 56300, Pakistan

**Keywords:** Reusable cement, Transformative construction, Marble powder recycling, Photo-chemical surface modification, MP surface chemistry

## Abstract

Sustainability and environmental protection are reshaping industries, including construction, where sustainability plays a crucial role in its influence on global resource consumption and waste management. The current study has developed a reusable cement material by photo-chemical surface modification of marble powder, achieved by reacting glycidyl methacrylate with carbonate functionality. This innovative modified marble powder boosts the reusability of construction materials, unlocking new possibilities for sustainable building practices. By transforming waste into valuable resources, we minimize raw material consumption, foster recycling, and advance circular economy principles in the construction industry. The difference in unmodified (22.9 nm) and modified (23–27 nm) marble powder particle sizes was used to assess the effectiveness of surface modification technique. Compared to non-reusable counterpart (20.34 MPa), the reusability has come at the cost of a decrease in compression stress (11.30 MPa, 10.89 MPa, and 10.57 MPa). Overall, photochemically modified marble powder for its reusability is a groundbreaking approach that offers substantial environmental, economical, and technical advantages, making it an appealing choice for the construction industry.

## Graphical abstract

**Figure:**
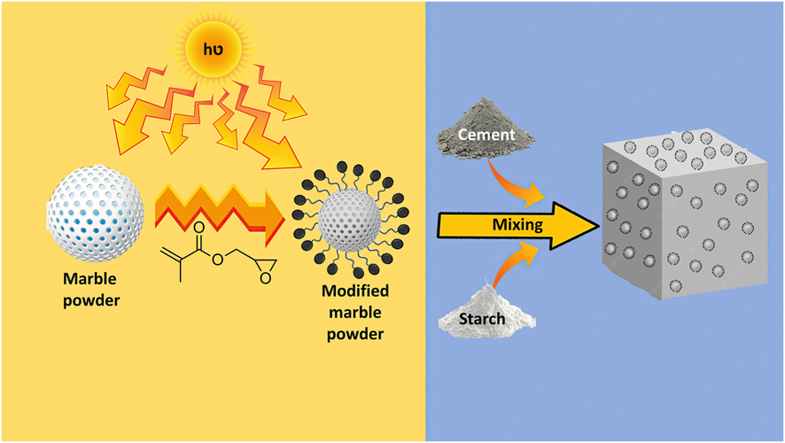

## 1 Introduction

Marble powder (MP) is a type of non-biodegradable debris that is linked to a range of environmental issues, such as soil porosity, permeability, and fertility decline [1]. Using MP in concrete production is a sustainable approach that can mitigate its negative environmental impact [2]. It is a common filler in construction industry [3], however, range of its applications widens after slight surface modification, from artificial stoneware [4] to air quality improving material [5]. MP in traditional building materials has been reported for 17 and 19 % improved compressive and tensile strength with 7.5 % MP in place of cement [6]. Another study [7] reported 18 % replacement of MP increased compressive strength by 37 % compared to neat sample.

Cement is usually applied once because crystal formation prevents remolding. Cement solidification happens in two steps, i.e., setting, an initial phase where cement paste turns into a gel, and hardening, the last phase where crystallization happens that imparts strength to the material [8]. In any instance, cement strength equates to gel and crystal formation due to the hydration of calcium aluminate [9,10] and hydrolysis of calcium silicate and calcium aluminate [10,11]. Formed calcium hydroxide transforms into needle-shaped crystals with in the colloidal gel, enhancing its strength. Aluminium hydroxide fills the gaps, leading to the solidification of the substance. The gel formed undergoes a process where it gradually loses water through evaporation, eventually solidifying into a hard mass.

The analysis of filler surface chemistry as a mechanical reinforcement in cement has been extensively studied [[12], [13], [14]]. However, there is currently no available data on the reusability of cement in relation to filler particularly MP. Nevertheless, there have been reports of sulfonated cement being used as reusable cement in atomic reactors [15].

A comparative analysis of all three types of materials has been done in Table 1 to emphasize the uniqueness of current research work.

**Table 1:** Comparison of existing research literature on MP based and reusable cements.

Cement	Filler	Working pH	Principal	Reconstruction Media	Ref
Ca-S-Al	Sand	Basic only (10–11.5)	Dissolution of individual components	Acid	[15]
Ca-Si-Al	Sand-MP, Sand-Si, Sand-metakaolin	NR	Non-Reusable	NA	[12]
Ca-Si-Al	MP, Epoxidized MP	Acidic-Neutral-Basic	Dissolution of crystals formed	Water	∗

• Current Study.

Historically, cement has been closely associated with durability, as once poured, it remains in position indefinitely. Nevertheless, the introduction of reusable cement contradicts this idea, presenting a fundamental change in construction approach. The current study uses construction material made by adding 80 wt % MP as filler without employing sand as a supporting filler. Previous studies used mixes of sand (major) and MP (minor) as fillers [[16], [17], [18], [19]]. This material's chemistry differs from that of previously documented reusable cements in that it doesn't contain any sulfur ingredients. Prepared samples exhibited both the structural integrity and longevity of traditional cement, while also introducing a unique concept: the reusability. Through this innovative approach waste material has been transformed into a valuable reinforcing agent.

## 2 Materials and method

### 2.1 Materials

MP was obtained from a local shop. Glycidyl methacrylate (GM) (DAEJUNG, Extra Pure), Triarylsulfonium hexafluorophosphate salt, mixed (THSM) (Sigma Aldrich, 50 %), Propylene carbonate (DAEJUNG, above 99.5 %) NaOH (Sigma Alrich, above 97 %), HCl (Merak, 37 %) and Oxalic acid (Sigma Aldrich, 99.5 %). Starch, cement, and sand were obtained from a local shop. Deionized water was used to polymerize the starch. UV lamp with 33 mW/cm^2^ intensity has been used as UV radiation source, in order to irradiate PI and subsequently start of the photochemical reaction.

### 2.2 Method

#### 2.2.1 Sample preparation

**Fig. 1:**
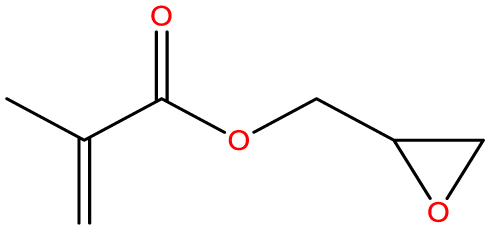
Structure of glycidyl methacrylate.

**Fig. 2:**
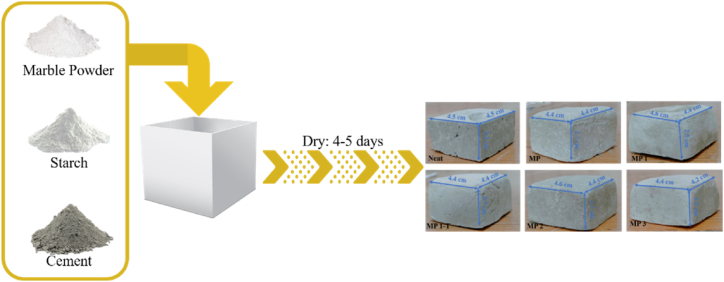
Schematic presentation of brick formation.

Air dried MP was passed through the sieves (≈125 mm) to obtain uniform particles. The surface chemistry of MP was enhanced through photo-chemical surface modification [20] using GM (Fig. 1). Modifier (GM) is a multi-functional molecule with two active functionalities, out of these, one is alkene and the other is epoxy. Both of these active functional groups respond to UV irradiated photoinitiator in photomechanical reactions. Various mole percentages of GM were utilized in the process (see Fig. 2).

Sample compositions were kept as per the data given in Table 2. Half of the modifier was sprayed initially, followed by a spray of 10 ml of PI solution (prepared using propylene carbonate). The mixture was then placed under a VU lamp for 10 min to allow the reaction to proceed. Then, the remaining half amount of the modifier and 10 ml of PI solution were sprayed, and the sample was placed under a UV lamp for 30 min. Then, modified MP samples were dried at 50 °C for a specific time.

**Table 2:** Photo-chemical surface modification of MP.

Sample ID	MP: Modifier (% Mole ratio)	PI (ml)	Irradiation Time (min)	Oven Drying Time (h)
MP	Nil	Nil	Nil	Nil
MP-1	1:0.45	20	40	48
MP-2	1:0.30	20	40	24
MP-3	1:0.15	20	40	24

### 2.3 Brick formation

Process involved polymerization of starch in hot water, followed by its combination with MP and cement in a weight ratio of 80:20. Mixture was then agitated for 10 min until it achieved homogeneity. The mixture was allowed to rest for 1 h before forming bricks. Brick MP-1-T was dried in an oven for 24 h at 70 °C to examine the impact of heating on the drying process; all other bricks were left at room temperature for four to five days. Sample casting was done in triplicate containing the same ingredients, under the same conditions, by using ASTM C 109. Standard (Neat brick) was also prepared with sand in place of MP in order to compare compressive strength.

### 2.4 Brick reconstruction

Serendipitously, while repairing fractured bricks, it was discovered that shattered pieces of bricks absorbed water and created a slurry suitable for reforming brick corners. This finding inspired a groundbreaking approach. Then entire brick was dismantled and rebuilt to check its mechanical strength. Photochemically surface modified waste MP was utilized in this experiment.

Once compressive strength was determined, crushed bricks, containing MP, were finely ground and mixed with water to create a paste. kept for a period and subsequently placed into a mold to achieve desired shape. Neat brick was created using conventional ingredients and established protocols. These ingredients have never been reported for potential reusability. To keep consistency in experimental protocols neat brick was also attempted for reconstruction, but attempt was unsuccessful, as the paste and material did not achieve adequate strength during molding, rendering reusable neat brick production unviable.

## 3 Characterization

A variety of tests were conducted to assess the properties of the prepared samples. Fourier Transform Infra-red (FTIR) absorbance of samples was done by using an “IR spirit Shimadzu instrument” with diamond crystal ATR, in 400–4000 cm^−1^, with a resolution of 16 and scan of 20.

Unmodified MP and MP1 have been analyzed for morphological changes through Field Emission Scanning Electron Microscope (FESEM; Tescan Lyra-3). Thermogravimetric analysis (TGA) was done by a Synchronous Thermal Analyzer (skz10600) in a working temperature range of 25–500 °C, with a heating rate of 10 °C per second. STA of all samples has been performed from RT to 500 ^O^C, where material stability has been comparatively analyzed on account of modifier. Thermal stability of MP has not been analyzed, rather instability in modifier structure or arrangement has been investigated. XRD analysis was done by using “TD-3500 X-ray Diffractometer” with Copper X-ray Source. The prepared sample's particle size was determined using the Scherrer equation (Eq i) [21].

Eq (1) D = K λ/β cos θ

Bragg's equation (Eq ii) was used to find the d-spacing [21].

Eq (2) d = nλ/2sinθ

Crystallinity % was determined by using Eq iii [22].

(iii) $$Crystallinity=\frac{Ares\;of\;crytalline\;peaks}{area\;of\;all\;peaks\;\left(crytalline+amorphous\right)}\times 100$$

pH and conductivity of samples were measured by “Milwaukie MW805 pH/EC/TDS/temperature meter”. For the analysis, 0.005 g of each sample was dissolved in 10 ml of deionized water and stirrer for 3 h and kept aside for 1 h to settle down. Base contents were determined by the direct titration method [23]. It involved weighing and adding 0.005 g of each sample into 25 ml of 0.01 M HCl solution, followed by shaking for 24 h and filtration. Then, the aliquant was titrated against 0.01 M NaOH solution to find the base contents using equation (iv).

Eq (iv) n_csf_ = (A ∗ V_a_ - B ∗ V_b_) ∗ V_a_/a

Where, n_csf_ = No of moles of basic functionalities on the carbon particles, A = strength of the acid, Va = volume of acid added, B = strength of the base, Vb = volume of base used, a = volume of aliquant taken.

The dispersion of 0.05g samples was checked into 10 ml distilled water. Time of settling was noted after stirring for 15 min.

Compression testing was performed by a digital machine (30 kN Electromechanical Universal Testing Machine, Jinan Scientific Test Technology Co. Ltd China) with a maximum load capacity of 30 kN and load accuracy ≤ ± 1 % from 2% to 100 % of full load.

The prepared sample was ensured to be defect free and then specimen's dimensions were recorded. After that, sample brick was positioned between the top and bottom grip assemblies of compression testing machine. Test setting was configured to test speed (1 N/min). The load cell detected applied force as the actuator pressed down on the specimen. Value was recorded when the sample started first sign of disintegration.

The test results were analyzed from compressive strength, measured in units of force per unit area, obtained by dividing the maximum load recorded by the machine by the specimen's cross-sectional area. A reported method [24] was used to measure the shear stress of prepared samples, as shown in equation (v).

Eq (v) Shear Stress = (0.707 × Applied Force)/ Area

Electrochemical analysis was performed by using Multimeter DT-830D at DCV mode in 20V range. Measured weight of sample lumps were placed on Aluminum foil and tested for electrochemical response in dry condition. Then samples were sprayed with small amount of water and once again tested for electrochemical response.

## 4 Results and discussion

### 4.1 FTIR (MP)

**Fig. 3:**
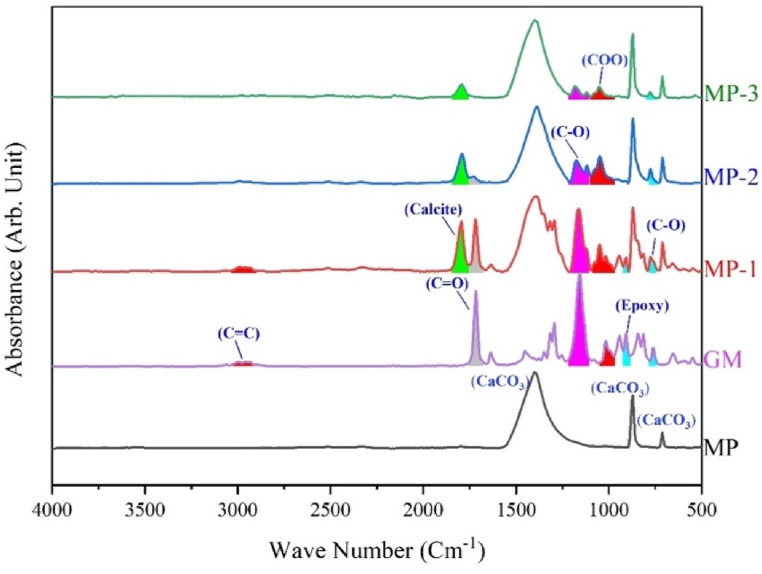
FTIR graph of unmodified and modified MP.

**Scheme 1:**
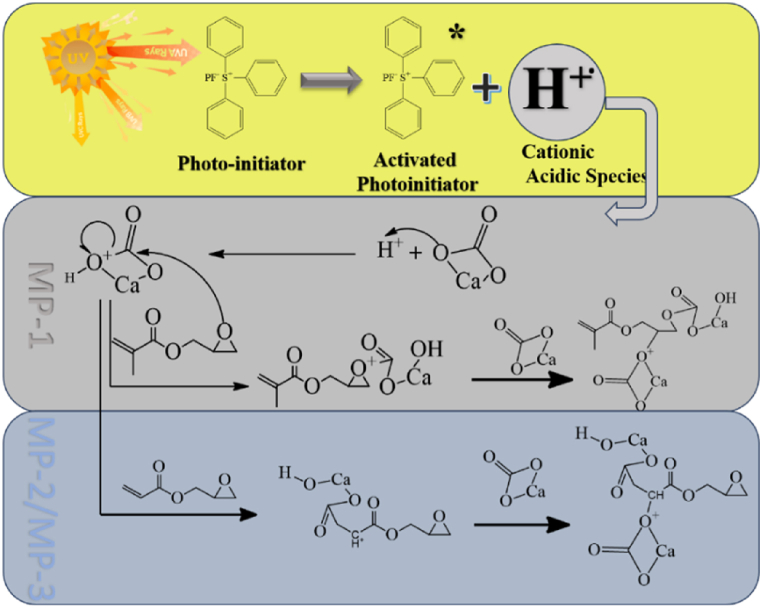
Proposed reaction mechanism for photo-chemical surface modification.

FTIR absorbance spectra of unmodified and modified MP have been presented in Fig. 3. Characteristic peaks of CaCO_3_ have been observed in an unmodified sample at 1407 cm^−1^ (asymmetric stretching of CO_3_^2−^), 871 cm^−1^ (out of plane CO_3_^2−^ bending), and 712 cm^−1^ (in plan CO_3_^2−^ bending) [25,26], whereas modified counterparts have been furnished with sharp peaks of Calcite at 1789 cm^−1^ and epoxy (C-O) at 774 cm^−1^. At 1048 cm^−1,^ an ester peak for glycidyl methacrylate has been observed. Ester characteristic peaks at 1179 cm^−1^ and 1116 cm^−1^ have been observed. Spectra of MP-2 and MP-3 have no C=C peak, indicating that the alkene of GM has been consumed during the surface modification of MP. But, MP-1 showed peaks of alkene and epoxy at 3000-2900 cm^−1^ and 774 cm^−1^, indicating modifier attachment with MP through both alkene and epoxy functionality. In UV modification, the alkene functionality is the sole anchor on the MP surface, while in thermal modification, the modifier can attach through the epoxy ring. In MP-1, a modifier is attached through alkene as shown in Scheme 1. Calcite and carbonyl group peaks are present at 1789 cm^−1^ and 1714 cm^−1^. FTIR results confirmed augmented surface functionalities on MP due to successful modification with GM. An upsurge in surface functionalities has imparted typical hydrophobic behavior.

#### 4.1.1 Proposed reaction mechanism

The FTIR data supports the predicted reaction pathway for the photo-chemical surface modification of MP-1 and MP2/3, as depicted in Scheme 1. FTIR study (Fig. 3) detected the existence of alkene functionality in MP1, however it was not found in MP2 and MP3.

### 4.2 SEM

**Fig. 4:**
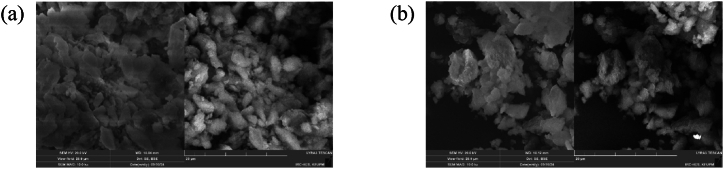
Sem: (a) Unmodified MP and (b) modified MP1.

SEM of both unmodified and modified MP has been performed to compare the morphological changes in the MP structure and its surface. The unmodified MP has a flakes-like structure with a smooth surface, clearly visible at different magnification levels (Fig. 4 (a). In contrast, the modified MP (MP-1) displayed an agglomerated morphology with quite a rough surface, providing unambiguous evidence of the successful surface modification of MP through the photochemical method, as illustrated in Fig. 4 (b). Both observations regarding rough surface and agglomerization can be attributed to modifier attachment onto MP surface. The multifunctionality of the modifier has led to several types of linkages with the MP (Scheme 1), hence facilitating surface roughness and agglomeration.

**Fig. 5:**
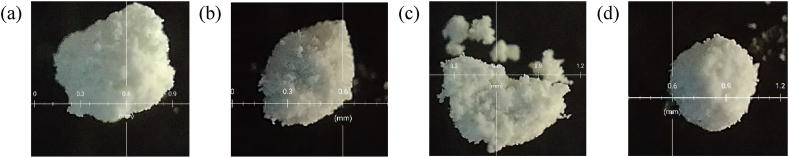
Microscopic image (a) MP, (b) MP-1, (c) MP-2 and (d) MP-3.

Microscopic images of unmodified and modified samples were taken to investigate agglomerate size (secondary aggregates), as presented in Fig. 5. Particles accumulate to form aggregates, and aggregates further accumulate to form agglomerates or secondary aggregates [27,28]. After modification, decreased agglomerate size of MP-1 and MP-3 has been observed, which might be attributed to overcrowded and compact modifier layers around aggregates, resulting in decreased agglomerization of MP particles. Conversely, with MP-2, a significant growth in agglomeration size has been observed, counter-confirming a well-organized modifier layer around each particle aggregate. This increase is caused by the highest connection of surface functions across aggregates.

### 4.3 Thermal analysis

**Fig. 6:**
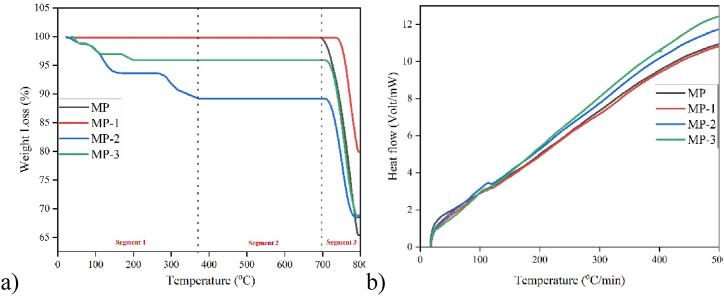
a) TGA and b) DSC of unmodified and Modified MP.

A comparative analysis of thermal stability for both unmodified and modified MP has been made by TGA (Fig. 6a), from room temperature to 800 °C. Thermal degradation patterns of all samples have shown three distinct segments in TGA spectra. MP and MP-1 exhibit comparable thermal behaviour, with the exception of temperatures exceeding 700 °C. MP, the unmodified sample, demonstrated thermal stability up to 700 °C, while MP-1, the modified sample, exhibited thermal stability up to 739 °C, underscoring the effectiveness of the surface modification protocol. Subsequently, a significant weight loss of up to 35 % in MP was noted as a result of the decomposition of CaCO_3_ into CaO and CO_2_ [29]. In contrast, MP-1 exhibited a maximum weight loss of 20 % in same temperature zone, indicating that modifier coating was causing difficulties in CaCO_3_ decomposition. Among all samples, both unmodified and modified, MP-1 has demonstrated the highest thermal stability, likely due to the compact material structure achieved after modification.

Among all modified samples, MP-2 exhibited the highest weight loss in the initial segment, likely due to desolvation, vaporisation, or the decomposition of volatile components [30]. Subsequently, MP-2 demonstrated thermal stability up to 710 °C, after which weight loss occurred again due to the decomposition of CaCO_3_. MP-3 showed same pattern of thermal instability. Both of these samples exhibited a phenomenon that can either be attributed to modifier's chemical composition (oxidizable constituents) [31] or its coating configuration (loosely packed modifier coating) [31]. According to the data presented in Fig. 6a, all modified samples in the third segment exhibited superior thermal stability compared to the unmodified material, likely due to the presence of the modifier coating on these samples.

Modified samples MP-2 and MP-3 showed better heat flow values (thermal dissipation), indicating superior thermal conductivity (Fig. 6b). However, a difference in their thermal stabilities (Fig. 6a) gives a clue about their structural variance [32]. Better thermal stability and improved thermal dissipation of MP-2 indicate an effective and even distribution of the modifier around the filler. The research findings indicated that lower modifier ratios resulted in more favorable outcomes when assessing thermal dissipation. The reduced thermal dissipation may be caused by the excessive presence of modifier molecules surrounding MP, leading to the formation of a densely packed layer with inadequate connection. The improved heat flow and thermal stability seen in sample MP-2 suggest that the modifier ratio used was appropriate for creating an effective modifier layer surrounding MP.

### 4.4 XRD

**Fig. 7:**
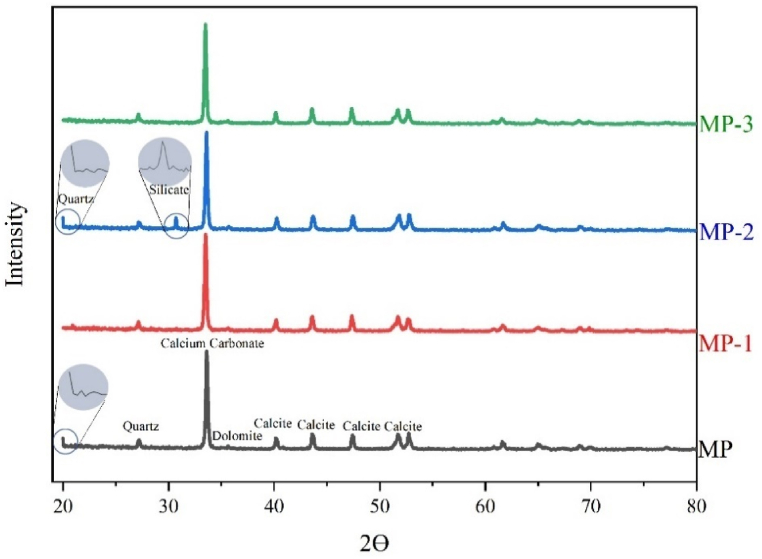
XRD graph of unmodified and modified powder.

XRD analysis (Fig. 7) revealed that the modified powder exhibited distinct and well-defined peaks, indicating its crystalline structure. XRD analysis of samples indicated that the minerals present in MP are calcium carbonate (CaCO_3_) and calcite (a more stable polymorph of CaCO_3_) in maximum quantities [33,34]. The presence of quartz (SiO_2_) and dolomite (CaMg(CO_3_)_2_) minerals is detected in minimal quantities [35], together with traces of Al_2_O_3_. In Mp-2, Silicate [33] minerals have been also detected.

Although the highest modifier ratio was present, a slight expansion in particle size of MP-1 indicated the formation of a dense modifier coating around MP (Table 4). On the other hand, despite having a relatively lower modifier ratio, a significant rise in particle size of MP-2 confirmed the formation of a well-structured modifier layer with improved molecular connections. A reduction in the size of MP-3 particles signifies a decline in the modifier's structural arrangement. The significant augmentation in the d-spacing of CaCO_3_ after modification indicates successfully incorporating a modifier between the particles. The observed rise in percentage crystallinity after the modification provides evidence of a well-organized arrangement of MP particles and modifier. MP-2 exhibited a maximum crystallinity of 77.37 %.

### 4.5 Physico-chemical analysis

Before assessing the prepared samples' surface chemistry, a preliminary evaluation was conducted using pH measurements, as indicated in Table 5. All samples exhibited a basic pH, indicating the presence of non-acidic functionalities on the surface of prepared samples. An analysis was conducted to determine the concentration of active basic components on the MP surface. Results confirmed a decrease in free surface functionalities upon modification (Table 5), evidencing utilization of functional groups during modification. MP2 has been observed for the least free surface functionalities (1.6478 mmol/g), confirming the development of maximum connections between MP and modifier molecules.

**Table 3:** Composition of bricks.

Sample ID	MP/Cement (g)	Starch (g)	Water (ml)	Drying Temp (°C)	Setting time (Days)
Neat Brick	80:20^a^	Nil	30	Rt	1
Brick MP	80:20	5	50	Rt	4–5
Brick MP-1	80:20	5	50	70	4–5
Brick MP-1-T	80:20	5	50	Rt	1
Brick MP-2	80:20	5	50	Rt	4–5
Brick MP-3	80:20	5	50	Rt	4–5

a Sand:Cement (No MP).

**Table 4:** Particle size d-spacing and % Crystallinity of Unmodified and modified MP.

Sample ID	Size (nm)	d spacing (Å)	Crystallinity (%)
MP	22.9	0.309763	69.3
MP-1	24.1	1.917577	71.8
MP-2	26.8	1.914377	77.4
MP-3	23.2	1.918192	74.2

**Table 5:** pH, Conductivity and base content of prepared samples.

Sample ID	pH	Conductivity (μS/cm)	n_csf_ (mmol/g)
Distilled Water	7.26	74	NT
MP	8.20	137	1.6878
MP-1	8.17	178	1.6614
MP-2	8.25	99	1.6478
MP-3	8.27	101	1.6614

### 4.6 Dispersion

**Fig. 8:**
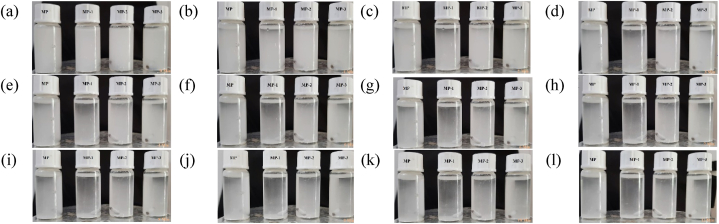
Dispersion of modified and unmodified sample.

Dispersion of unmodified and modified samples in distilled water is presented in Fig. 8. A poor dispersion for MP-1 indicates a compact and densely packed material that settles down easily. Although the functionalities of MP-1 are higher than those of MP-2 (data from Table 5), the overcrowding of surface functionalities resulted in hydrophobic conduct and subsequently reduced dispersion.

## 5 Bricks analysis

Given that the material was specifically engineered for construction applications, samples were employed to fabricate bricks by including them in a standardized mixture of sand and cement. In certain formulations, sand has been substituted with unmodified or modified MP samples. Composition detail is mentioned in Table 3.

### 5.1 Physico-chemical analysis

pH and conductivity analyses were done for all samples (Table 6). A slight increase in both pH and conductivity was noted in unmodified MP brick material upon comparison with neat brick counterpart. On the contrary, decreased pH and conductivity values were seen in modified samples with reference to unmodified MP bricks (with the exception of MP-1-T). The reduced conductivity indicates a decline in the movement of ions. This, in turn, corroborated the rate of hydride production and product stiffening.

**Table 6:** pH and conductivity of bricks.

Sample ID	pH	Conductivity(μS/cm)
Neat Brick	9.7	211
Brick MP	9.9	243
Brick MP-1	9.0	238
Brick MP-1-T	8.7	252
Brick MP-2	9.2	208
Brick MP-3	9.1	228

### 5.2 FTIR analysis

**Fig. 9:**
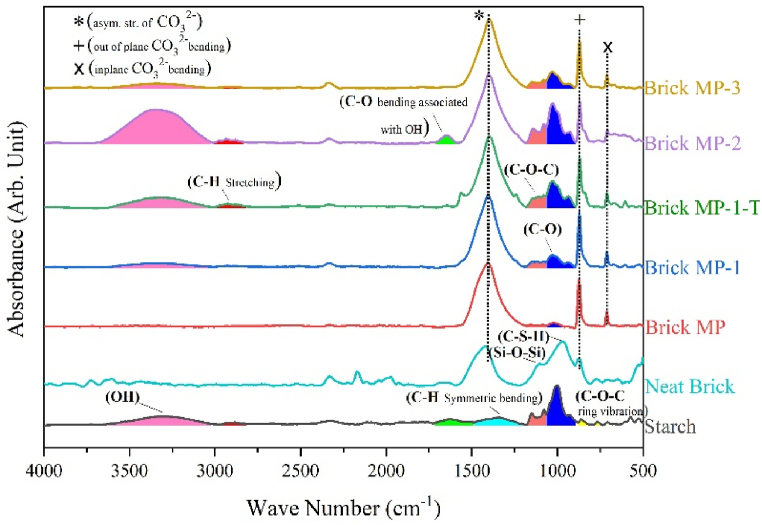
FTIR Graphs of prepared brick samples.

FTIR of starting materials and products are shown in Fig. 9. FTIR of neat brick revealed characteristic peaks for CO_3_^2−^ at 871 (out of plane CO_3_^2−^ bending) and 1407 (asymmetric stretching of CO_3_^2−^) [36], alongwith calcium silicate hydrate (C-S-H) and Si-O-Si pekas at 980 cm^−1^ and 1100 cm^−1^, respectively [37,38]. Starch spectra showed board peak of OH stretching at 3300-3600 cm^−1^, C-O bending (associated to OH) at 1635 cm^−1^ [27,39], C-O-C asymmetric stretching at 1150 cm^−1^ and C-O stretching at 1002 cm^−1^ [27,28,40]. In starch C-O-C ring vibrations were also observed at 860 cm^−1^ and 769 cm^−1^ [41]. In all prepared brick samples, characteristic peaks of CaCO_3_ at 1407 cm^−1^, 871 cm^−1^, and 712 cm^−1^ were observed [25,26], alongwith C-H stretching (2928 cm^−1^) and C-H symmetric bending (1330-1400 cm^−1^) [41]. In MP bricks, C-O and C-O-C peaks are totally different to distinct peaks of Si-O-Si and C-H-S in neat brick, indicating formation of different products. It has also been observed from the setting time of brickes (data in Table 3) that modified MP influenced reaction kinetics and cement hydration. FTIR data demonstrated that MP-based samples, upon reaction between components, formed entirely distinct connections compared to sand-based neat brick.

### 5.3 Thermal analysis

**Fig. 10:**
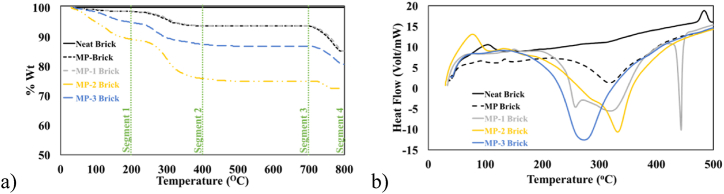
a) TGA and b) DSC of bricks.

Thermogravimetric analysis (TGA) was employed to assess thermal stability of prepared bricks (Fig. 10a). All samples presented four segments in thermal degradation patterns, out of which three showed weight loss due to dehydration [42], change of dolomite into calcite [43], and decomposition of CaCO_3_ [29]. Decrease in weight from 100 to 300 °C relates to dehydration [42]. MP weight loss up to 400 °C has been attributed to transformational crystallization of dolomite into calcite [43], whereas MP weight loss between 700 and 790 °C has been reported for decomposition of CaCO_3_ [29]. Upon comparison, neat brick demonstrated the best thermal stability. Both MP and MP-1 bricks exhibited analogous degradation patterns, with MP-1 brick demonstrating marginally superior heat stability in segments relating to transformational crystallization of dolomite into calcite and CaCO_3_ decomposition, potentially due to the modifier coating on MP. MP-2 brick displayed the greatest weight loss, while MP-3 brick showed the second highest level of thermal instability. This may be associated with the smaller particulate size of MP-3, which integrated effectively with other components in the brick, while 77 % crystallization of MP-2 may have caused a micro phasic separation among the brick elements.

Heat changes were measured using DSC, as illustrated in Fig. 10b. In MP, MP-2, and MP-3 bricks endothermic peaks were seen at 317 °C, 272 °C, and 330 °C, respectively. These observations advocate the change in heat flow due to transformation of dolomite into calcite [43], which is the most stable form. Significant heat change has been observed in these regions as shown in Fig. 10a. Two endothermic peaks at 257 °C and 321 °C were found in MP-1 brick, indicating the conversion of dolomite to calcite. An endothermic peak at 440 °C was observed, which suggests the release of acrolein from MP-1 brick [44]. These findings counter confirm FTIR observations for MP-1 (Fig. 3), which showed the presence of alkene and carbonyl groups. The proposed reaction mechanism (Scheme 1) also supports this conclusion.

### 5.4 Electrochemical response

An exceptionally unique reaction of prepared bricks has been observed in terms of their electrochemical behavior (Table 7). When linked to aluminum foil using a voltmeter, the bricks displayed distinct voltage levels. This distinctive electrochemical reaction indicated a particular inclination of the prepared bricks to generate a specific current when connected to aluminum foil. The analysis was conducted under two distinct conditions: when the bricks were dry and when they were wet. In dry conditions, only bricks made with modified MP showed an electrochemical response. However, under wet conditions, every sample exhibited an electrochemical reaction. The Brick MP-2 demonstrated the highest electrochemical reaction of approximately 0.3V when dry and 1.0V when wet. It is important to note that a standard AA alkaline battery typically provides a voltage of 1.5 V.

**Table 7:** Electrochemical response of bricks.

Sample ID	Weight (g)	Electrochemical (V)	
		Dry state	Wet state
Neat Brick	1.31	0.00	0.90
Brick MP	2.91	0.00	0.80
Brick MP-1	2.03	0.20	0.50
Brick MP-1-T	2.46	0.01	1.00
Brick MP-2	2.53	0.30	1.00
Brick MP-3	2.18	0.00	0.90

### 5.5 Compression strength

**Fig. 11:**
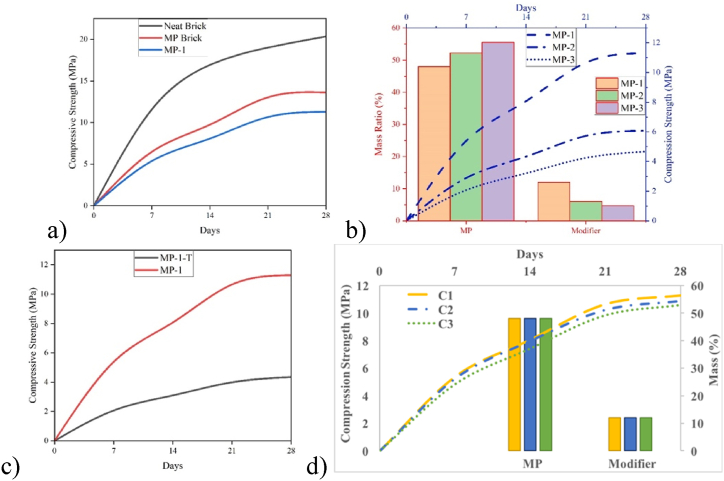
Compressive strength of a) Neat, MP and MP-1 brick samples; b) Modified brick samples; c) MP-1 bricks cured at different temperatures; d) MP-1 cycles.

Fig. 11a–c displays the compression strength of all samples over time, demonstrating a positive correlation between the age of bricks and their compressive strength. The samples achieved their maximum strength after 28 days, when compact crystals were formed. After 28 days, the neat brick showed a maximum compression strength of 20.34 MPa (Fig. 11a), whereas the unmodified MP brick, without sand, had a maximum compression strength of 13.16 MPa, which is approximately 60 % of the strength of a regular brick. Among all the modified MP brick samples, MP-1, which was manufactured at room temperature, exhibited the maximum compression strength of 11.30 MPa, nearly half of the compression strength of the neat brick. The compression strength of thermally dried Brick MP-1-T was considerably lower than that of the brick dried at room temperature (Fig. 11c), suggesting that thermal drying does not contribute to mechanical strength development. Brick MP-2 demonstrated compression strength of 6.07 MPa, whereas Brick MP-3 revealed compression strength of 4.67 MPa (Fig. 11b). The correlation between compressive strength and MP:Modifier ratio was found to be positive, indicating that a rise in the modifier ratio led to an increase in compressive strength, and vice versa. However, modified MP bricks still exhibited lower strength than the unmodified version. Subsequently, neat, unmodified MP and MP-1 bricks were meticulously pulverized and mixed with water to generate a slurry, which was then utilized to rebuild the bricks. Only MP-1 brick material exhibited a quantifiable level of strength during reconstruction (1st cycle 11.3 Mpa, 2nd cycle 10.89 Mpa and 3rd cycle 10.57 MPa at 28 days). This process was repeated once more. The compression strength of MP-1 bricks was evaluated to be identical in all three cycles of demolition and reconstruction (Fig. 11d).

### 5.6 Shear stress

**Fig. 12:**
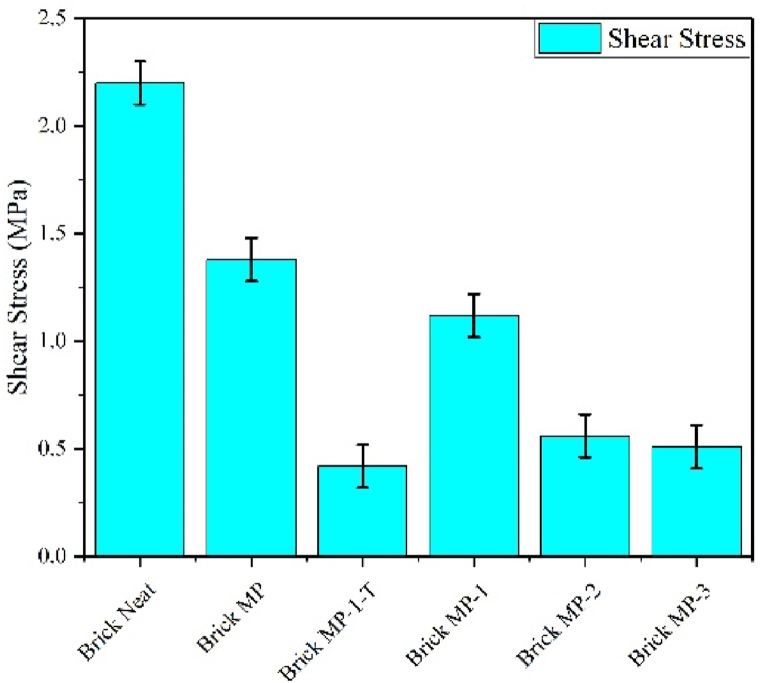
Shear stress of brick samples.

The utilization of photochemically modified marble powder as a reusable construction material offers novelty and brings about innovation and advantages in terms of sustainable material usage, enhanced material properties, cost-effectiveness, eco-friendly construction, reusability and recycling, and versatility in applications. Maximum shear stress was calculated by using equation (iv), and data is represented in Fig. 12.

## 6 Conclusion

MP has been converted from waste into a reusable material via a facile photo-chemical approach that modified its surface chemistry, resulting in a construction material with quantifiable mechanical strength. Augmented hydrophobicity has affected cement setting reactions without damaging the material's mechanical strength. FTIR confirmed difference in hydration products of MP based samples in comparison to neat brick (sand based sample). MP modification has reduced shear stress from 13.61 MPa (unmodified MP) to 11.3 MPa (modified MP), yet this drop in shear stress has been offset by the product's reusable nature. Modified MP based material has been rebuilt three times with roughly the same mechanical strength. In contrast to traditional cement, basically reusable cement offers the benefits of sustainability and decreased environmental impacts, albeit at the expense of mechanical strength.

## 7 Recommendation

Unequal amount of water in both (sand based and MP based) recipes indicated inconsistency in cement hydration process. Modifier influenced cement crystal formation, enabling possibility of brick reconstruction. Time interval to complete hydration process was different for both bricks, i.e. brick containing sand achieved setting in one day, while MP containing bricks did not properly set before 4–5 days. Considering this, authors cannot conclusively state the effect of modified marble waste on cement hydration process. However, reusability factor indicated that modified marble waste has influence reaction kinetics (from setting time difference), or hydration product formation (from FTIR data).

Further enhancement to optimize reusable cement performance, research should focus on developing novel formulations with a balance of enhanced mechanical strength and sustainable environmental benefits, mitigating the current trade-off between reusability and strength.

## CRediT authorship contribution statement

**Ali Zia Noor:** Writing – original draft, Methodology, Investigation, Formal analysis, Data curation. **Muhammad Atif:** Writing – review & editing, Writing – original draft, Supervision, Resources, Methodology, Investigation, Conceptualization. **Sadia Bibi:** Writing – original draft, Investigation. **Muhammad Burhan Sharif:** Writing – original draft, Resources, Investigation, Formal analysis. **Amjad Ali:** Writing – original draft, Investigation, Formal analysis.

## Data availability statement

Data that supports the findings of this study is available from the corresponding author upon reasonable request.

## Declaration of competing interest

The authors declare that they have no known competing financial interests or personal relationships that could have appeared to influence the work reported in this paper.
